# Effectiveness Study of Paromomycin IM Injection (PMIM) for the Treatment of Visceral Leishmaniasis (VL) in Bangladesh

**DOI:** 10.1371/journal.pntd.0004118

**Published:** 2015-10-23

**Authors:** Kazi M. Jamil, Rashidul Haque, Ridwanur Rahman, M. Abul Faiz, Abu Toha Md. Rezwanul Haque Bhuiyan, Amresh Kumar, Syed Misbah Hassan, Heather Kelly, Pritu Dhalaria, Sonali Kochhar, Philippe Desjeux, Mohammad A. A. Bhuiyan, Mohammed M. Khan, Raj Shankar Ghosh

**Affiliations:** 1 Kuwait Institute for Scientific Research, Environment and Life Sciences Research Center, Food and Nutrition Program, formerly International Center for Diarrheal Disease Research, Bangladesh (icddr,b), Mohakhali, Dhaka, Bangladesh; 2 International Center for Diarrheal Disease Research, Bangladesh (icddr,b), Mohakhali, Dhaka, Bangladesh; 3 Shaheed Suhrawardy Medical College Hospital (SSMCH), Sher-E-Bangla Nagor, Dhaka, Bangladesh; 4 Dev Care Foundation, Dhaka, Bangladesh and Retired Ministry of Health, Government of Bangladesh Official; 5 PATH, formerly OneWorld Health; 6 International AIDS Vaccine Initiative, formerly OneWorld Health; 7 John Snow India, formerly OneWorld Health; 8 Bill & Melinda Gates Foundation, formerly PATH; London School of Hygiene and Tropical Medicine, UNITED KINGDOM

## Abstract

**Background:**

This study was conducted in Bangladeshi patients in an outpatient setting to support registration of Paromomycin Intramuscular Injection (PMIM) as a low-cost treatment option in Bangladesh.

**Methodology:**

This Phase IIIb, open-label, multi-center, single-arm trial assessed the efficacy and safety of PMIM administered at 11 mg/kg (paromomycin base) intramuscularly once daily for 21 consecutive days to children and adults with VL in a rural outpatient setting in Bangladesh. Patients ≥5 and ≤55 years were eligible if they had signs and symptoms of VL (intermittent fever, weight loss/decreased appetite, and enlarged spleen), positive rK39 test, and were living in VL-endemic areas. Compliance was the percentage of enrolled patients who received 21 daily injections over no more than 22 days. Efficacy was evaluated by initial clinical response, defined as resolution of fever and reduction of splenomegaly at end of treatment, and final clinical response, defined as the absence of new clinical signs and symptoms of VL 6 months after end of treatment. Safety was assessed by evaluation of adverse events.

**Principal Findings:**

A total of 120 subjects (49% pediatric) were enrolled. Treatment compliance was 98.3%. Initial clinical response in the Intent-to-Treat population was 98.3%, and final clinical response 6 months after end of treatment was 94.2%. Of the 119 subjects who received ≥1 dose of PMIM, 28.6% reported at least one adverse event. Injection site pain was the most commonly reported adverse event. Reversible renal impairment and/or hearing loss were reported in 2 subjects.

**Conclusions/Significance:**

PMIM was an effective and safe treatment for VL in Bangladesh. The short treatment duration and lower cost of PMIM compared with other treatment options may make this drug a preferred treatment to be investigated as part of a combination therapy regimen. This study supports the registration of PMIM for use in government health facilities in Bangladesh.

**Trial Registration:**

ClinicalTrials.gov identifier: NCT01328457

## Author Summary

Effective and safe therapies for visceral leishmaniasis (VL), also known as kala-azar, at an affordable cost are urgently needed in Bangladesh. Although sodium stibogluconate, miltefosine, and paromomycin are included in the National Essential Drug List for VL, only miltefosine is currently registered in Bangladesh. Paromomycin is a low-cost drug that was demonstrated to be an efficacious and safe treatment for VL in endemic areas of India, which led to approval of paromomycin IM injection (PMIM) for the treatment of VL in India in 2006 and inclusion of PMIM in the WHO Essential Medicines List in 2007. A subsequent Phase IV trial in India revealed a similarly high efficacy and safety profile coupled with high treatment compliance in a rural outpatient setting. We confirmed the effectiveness, safety, and high compliance of PMIM when used in an outpatient setting in Bangladesh. This study supports the registration of PMIM as a low-cost treatment option for VL in Bangladesh.

## Introduction

Visceral leishmaniasis (VL), also known as kala-azar, remains one of the most neglected diseases in Bangladesh with an estimated 65 million people at risk of the disease [1]. Widely available, safe, and affordable therapies for visceral leishmaniasis are needed. Although VL was nearly eliminated in Bangladesh during the Malaria Eradication Programme of 1961–1970 [2], the country experienced an epidemic in the mid-2000s (9,379 cases reported in 2006 by the Ministry of Health and Family Welfare [MoHFW]) [3]. More recently, the number of VL cases reported by MoHFW has declined annually from 4,932 cases in 2007 to 3,300 cases in 2011 [3, MoHFW personal communication]. Although the decline in reported VL cases is promising, sustained efforts are needed to meet the Kala-Azar Elimination Program target of <1 case annually per 10,000 population at the upazila level by 2015.

VL mostly occurs in the poorest of the poor, whose poverty is further impacted by lost productivity when a family member is affected. Cases are treated for free in the Upazila Health Complexes, which are the government-run primary health care centers situated in the upazila (sub-district). Patients are referred to tertiary hospitals when they need evaluation for treatment failure, relapse, or other complications.

In Bangladesh, the transmission of VL is purely anthroponotic. Therefore, breaking the human reservoir host-vector cycle requires timely diagnosis and treatment of VL patients, a decrease in the human host reservoir, and rigorous reduction in vector contact with humans. The MoHFW of Bangladesh has instituted a kala-azar elimination program, including diagnosis and adequate treatment of all cases of VL, disease surveillance, and vector surveillance and control.

Although sodium stibogluconate, miltefosine, and paromomycin are included in the National Essential Drug List for VL, only miltefosine is currently registered in Bangladesh. Paromomycin is a low-cost drug that was demonstrated to be efficacious and generally safe and well tolerated for the treatment of VL in a Phase III trial conducted in endemic areas of India [4]. Based on results of the Phase III trial in India, paromomycin IM injection (PMIM) was approved for the treatment of VL in August 2006 by the Drugs Controller General of India and was included in the World Health Organization (WHO) Essential Medicines List in 2007. A subsequent Phase IV trial in India revealed a similarly high efficacy and safety profile coupled with excellent treatment compliance in a rural outpatient setting [5]. A clinical trial on different treatment regimens for VL completed in four East African countries showed that the combination of sodium stibogluconate (SSG) and PMIM administered over 17 days had efficacy and safety comparable with a 30-day course of SSG alone [6]. Thus, adding PMIM to the treatment regimen lowered both the cost and duration of treatment. This Phase IIIb study was designed to confirm the effectiveness of PMIM to treat VL in Bangladeshi patients in an outpatient setting to support registration of this low-cost treatment option in Bangladesh.

## Methods

### Ethics Statement

This study was conducted in accordance with the Bengal Drugs Rules, 1946 (as amended in 1952), the Drugs Act 1940 of the People's Republic of Bangladesh (as modified in 1964), the Declaration of Helsinki (adopted at the 59 World Medical Association General Assembly, Seoul, October 2008), and International Conference on Harmonization of Technical Requirements for Registration of Pharmaceuticals for Human Use—Good Clinical Practice (ICH-GCP). The research protocol (see Protocol S1) was approved by the Research Review Committee and the Ethical Review Committee of International Centre for Diarrhoeal Disease Research, Bangladesh (icddr,b); the Director General Health Services (DGHS), Bangladesh. icddr,b, also appointed an independent Data Safety Monitoring Board to monitor safety during the trial.

The trial is registered at ClinicalTrials.gov (identifier: NCT01328457). Written informed consent was obtained in accordance with ICH-GCP, the Declaration of Helsinki, laws and regulations of Bangladesh and all applicable regulatory requirements. If a patient was younger than 18 years old, his/her legal representative (a parent or legal guardian) signed the informed consent for the patient. Assent from the participating minor was also obtained. For illiterate patients, the entire informed consent procedure was witnessed by an impartial, literate person who attested (with his/her dated signature) that the patient gave informed consent voluntarily and that the information provided was a complete and accurate representation of the informed consent form. The Ethical Review Committee allowed illiterate patients to provide a thumbprint on the informed consent document. The written informed consent was signed and dated by subject/parents/legal guardian/witness as well as the investigator prior to any study-related procedure. Participation was voluntary, and patients could withdraw from the trial at any time without further obligation.

Rescue medication was available to patients who discontinued treatment with PMIM or who were considered a treatment failure.

### Study Design and Medication

This Phase IIIb, open-label, multi-center, single-arm trial was designed to assess the effectiveness and safety of PMIM (Gland Pharma Ltd., Hyderabad, India) administered at a dose of 11 mg/kg (paromomycin as the base) intramuscularly once daily for 21 consecutive days to children and adults with VL in rural Bangladesh. The planned sample size of 120 patients with VL was not based on statistical calculations but was considered a sufficient size to determine effectiveness and safety of PMIM in the Bangladeshi population based on high efficacy rates reported in large-scale studies of PMIM in Indian populations [4,5]. The study was conducted between January 2011 and June 2012 at two health complexes in Mymensingh District, Bangladesh. Because this was the first time that paromomycin was used in Bangladesh, the study team ensured supply of all equipment and materials. All clinical staff involved in the study received training on ICH GCP, study protocol activities including consent procedures, universal safety precautions, administration of PMIM, and management and reporting of adverse events.

### Case Definition and Study Population

Patients who were ≥5 years (weighing at least 5 kg) and ≤55 years old were eligible if they met the following case definition criteria: (1) living in the VL-endemic areas in Bangladesh, (2) signs and symptoms of VL including history of intermittent fever for at least 2 weeks, history of weight loss and/or decrease in appetite, and enlarged spleen, (3) a positive serological rK39 test. For patients who had a prior history of VL (and, therefore, anticipated positive rK39 results independent of current VL infection status), the clinical signs and symptoms and VL-endemic residency ultimately determined eligibility. Patients had to be clinically stable and able to maintain adequate hydration. Exclusion criteria included pregnancy or lactation; active tuberculosis or taking antituberculosis medications; previous treatment with PMIM; clinically significant anemia; current or history of clinically significant renal or hepatic dysfunction; serum creatinine above the upper limit of normal range; proteinuria; history of hepatitis B or C or HIV positive; history of hearing loss; significant coexisting disease; any history of VL or treatment for VL; history of hypersensitivity to aminoglycosides or sulfite; and concomitant use of other aminoglycosides, nephrotoxic and ototoxic drugs, or immunosuppressive drugs.

### Study Procedures

Enrolled patients received PMIM administered at a dose of 11 mg/kg (paromomycin as the base; dosing based on screening body weight) intramuscularly into the gluteus muscle once daily for 21 consecutive days (or over 22 days if one day was missed). Treatment and study assessments were conducted at the study center on an outpatient basis; however, patients who did not have access to local lodging could have been admitted to the study center facility. Safety was assessed throughout the study. Patients were assessed for initial clinical response on the last day of treatment (Day 21/22; end of treatment [EOT]); they were instructed to return to the study center if they had any symptoms of relapse, adverse event, or hearing loss within 30 days after EOT. Patients were not required to return for follow-up, but were encouraged to return if problems arose during this 30-day window. Six months after EOT (approximately Study Day 202), patients were asked to return for an assessment of final clinical response.

Patients could be removed from treatment if they experienced a life-threatening adverse event considered related to PMIM; reported hearing loss, tinnitus, or other unexplained auditory or vestibular symptoms; needed treatment with an immunosuppressant or a medication with ototoxic and/or nephrotoxic potential; or treatment failure (no improvement in disease severity after ≥14 days of PMIM treatment).

### Assessments

#### Compliance

Compliance was analyzed using the ITT population, which was defined as the percentage of enrolled patients who received 21 daily injections over no more than 22 days.

#### Efficacy

All enrolled patients were included in the ITT population for efficacy analyses. Initial clinical response was defined as the resolution of fever and the reduction of splenomegaly by palpation at EOT. Final clinical response was defined as the absence of new clinical signs and symptoms of VL at 6 months after EOT. Specifically, clinical response was evaluated in the following binary fashion: (a) Is the patient’s temperature less than 99.4°F in clinic at EOT visit (Yes/No); (b) Has the patient reported resolution of fever and no fever within the last 5 days? (Yes/No); (c) Has spleen size decreased from screening value? (Yes/No); (d) Is the clinical impression of the treating physician that of an adequate clinical response? (Yes/No). A clinical response was achieved if at least 2 of 3 answers to a, b, c were “Yes” and d was “Yes.”

Subgroup analyses based on age, sex, and study site were conducted; however, formal comparisons between subgroups were not conducted.

#### Safety

Safety was assessed by evaluation of all adverse events, regardless of causality, and vital signs. All patients who received at least one dose of PMIM were included in the Safety population.

Data on occurrences of PKDL cases after end of treatment were not collected.

## Results

### Study Cohort

**Fig 1:**
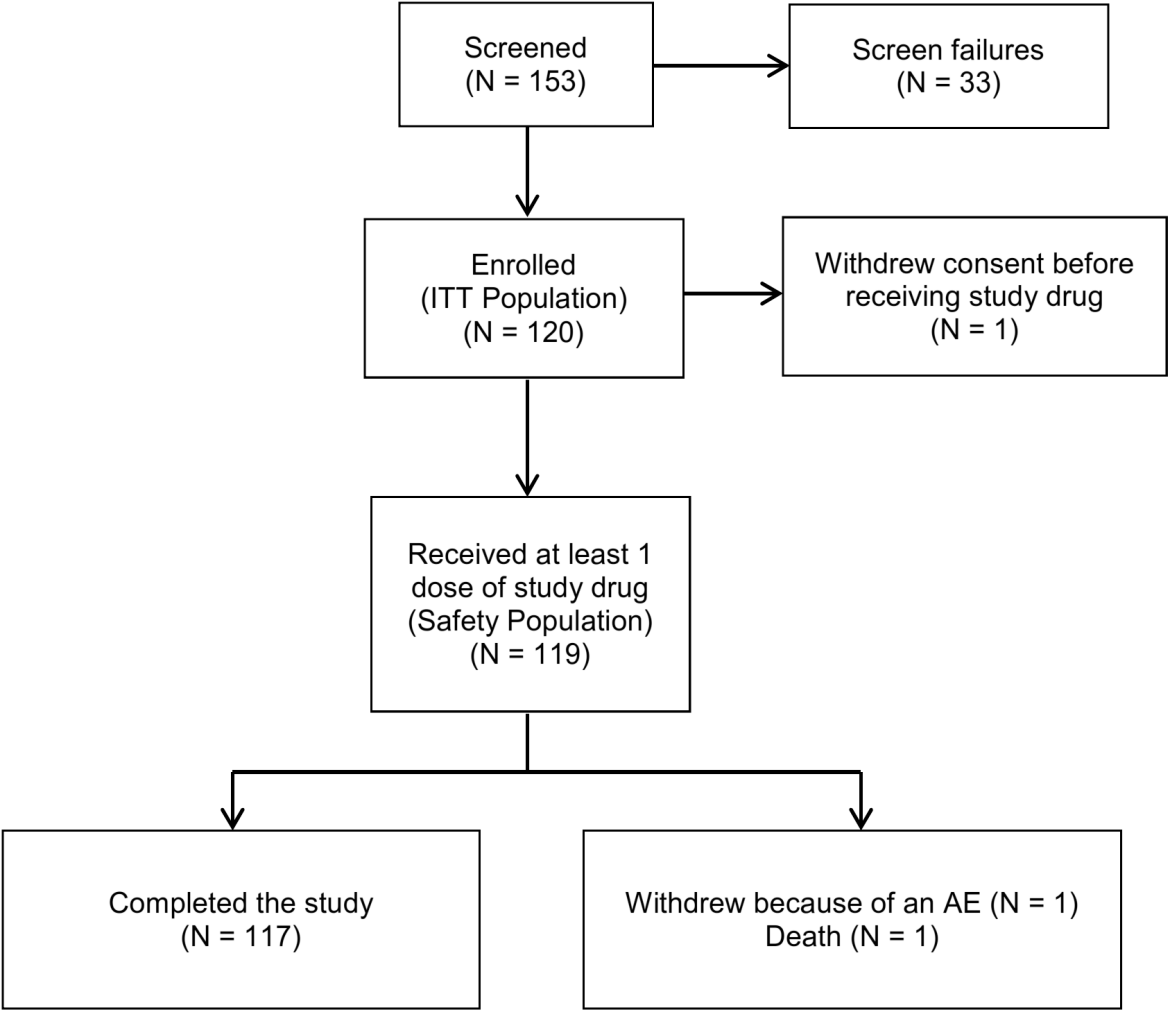
Patient disposition flow chart.

One hundred fifty-three children and adults were screened, of which 120 patients were enrolled and included in the intent-to-treat (ITT) population (Fig 1). A total of 117 subjects completed the study as per protocol (1 subject withdrew consent before receiving study drug and was excluded from the Safety population; 1 subject had a serious adverse event [SAE], and 1 subject died [described below in Safety Results]).

### Baseline Characteristics

Of the 120 enrolled subjects, 58% were male and 49% were pediatric (Table 1). The mean (± standard deviation) age was 19.4 ± 11.55 years (median 18; range 5–50).

**Table 1:** Distribution of patients by age and sex.

		ITT Populations	Safety Populations
Category	Subcategory	(N = 120)	(N = 119)
Age category, n (%)	Pediatric (2 to <18 years)	59 (49.2%)	58 (48.7%)
	Adult (≥18 to 55 years)	61 (50.8%)	61 (51.3%)
Sex, n (%)	Male	70 (58.3%)	70 (58.8%)
	Female	50 (41.7%)	49 (41.2%)

### Treatment Compliance

A total of 118/120 subjects (98.3%) were compliant with treatment (21 daily injections over no more than 22 days).

### Efficacy Results

The initial clinical response rate at EOT in the ITT population was 98.3% (95% confidence interval [CI] 96.0–100.6) (Table 2). Nearly all pediatric (58/59; 98.3%) and adult (60/61; 98.4%) subjects achieved initial clinical response. All males (70/70) and 96.0% (48/50) of females achieved initial clinical response.

**Table 2:** Efficacy results (ITT population).

	All Subjects	Sex Subgroups		Age Subgroups		Study Site Subgroups	
		Male	Female	Pediatric	Adult	Site 1	Site 2
Analysis	n (%)	n (%)	n (%)	n (%)	n (%)	n (%)	n (%)
Initial clinical response^a^	118/120 (98.3)	70/70 (100)	48/50 (96.0)	58/59 (98.3)	60/61 (98.4)	79/80 (98.8)	39/40 (97.5)
Final clinical response^b^	113/120 (94.2)	66/70 (94.3)	47/50 (94.0)	56/59 (94.9)	57/61 (93.4)	74/80 (92.5)	39/40 (97.5)

^a^ Initial clinical response was defined as the resolution of fever and the reduction of splenomegaly by palpation at EOT.

^b^ Final clinical response was defined as the absence of new clinical signs and symptoms of VL at 6 months after EOT.

The final clinical response rate 6 months after EOT was 94.2% (95% CI 90.0–98.4); 94.9% in pediatric (56/59) and 93.4% in adult (57/59) subgroups; and 94.3% (66/70) in males and 94.0% (47/50) in females.

### Safety Results

Of 119 subjects who received at least one dose of study drug, 118 subjects received 21 consecutive daily doses of the study drug, and one subject (adult female) had an SAE (ear infection requiring antibiotics) and was discontinued from study drug after receiving 4 doses of PMIM.

Adverse events are summarized in Table 3 and detailed in Table 4. Three severe adverse events were reported in two subjects who also had serious adverse events as described below.

**Table 3:** Summary of adverse events.

			Sex Subgroups		Age Subgroups		Study Site Subgroups	
		All Subjects	Male	Female	Pediatric	Adult	Site 1	Site 2
		(N = 119)	(N = 70)	(N = 49)	(N = 58)	(N = 61)	(N = 80)	(N = 39)
Category	Type of Adverse Event	n (%)	n (%)	n (%)	n (%)	n (%)	n (%)	n (%)
Adverse events, n (%)	Any adverse event^a^	34 (28.6)	17 (24.3)	17 (34.7)	15 (25.9)	19 (31.1)	24 (30.0)	10 (25.6)
	Severe or life-threatening^b^	2 (1.7)	1 (1.4)	1(2.0)	0	2 (3.3)	1 (1.3)	1 (2.6)
	Led to premature discontinuation of study drug	1 (0.8)	0	1(2.0)	0	1 (1.6)	1 (1.3)	0
	Serious, including events leading to death	4 (3.4)	2 (2.9)	2 (4.1)	1 (1.7)	3 (4.9)	3 (3.8)	1 (2.6)
Death, n (%)		1 (0.8)	0	1(2.0)	0	1 (1.6)	1 (1.3)	0

The Safety population includes all subjects who received ≥1 dose of study drug. Subjects with multiple occurrences of the same event are counted only once.

^a^ One event of pyrexia reported in an adult male at site 2 was not treatment emergent.

^b^ Grade 3 or 4 according to Cancer Therapy Evaluation Program Common Terminology Criteria for Adverse Events (CTEP-CTCAE) definition.

**Table 4:** Adverse events.

			Sex Subgroups		Age Subgroups		Study Site Subgroups	
		All Subjects	Male	Female	Pediatric	Adult	Site 1	Site 2
		(N = 119)	(N = 70)	(N = 49)	(N = 58)	(N = 61)	(N = 80)	(N = 39)
System Organ Class	Preferred Term	n (%)	n (%)	n (%)	n (%)	n (%)	n (%)	n (%)
No. of subjects with at least one AE		34 (28.6)	17 (24.3)	17 (34.7)	15 (25.9)	19 (31.1)	24 (30.0)	10 (25.6)
Blood and lymphatic system disorders		1 (0.8)	1 (1.4)	0	0	1 (1.6)	0	1 (2.6)
	Anaemia	1 (0.8)	1 (1.4)	0	0	1 (1.6)	0	1 (2.6)
Ear and labyrinth disorders		2 (1.7)	1 (1.4)	1 (2.0)	0	2 (3.3)	1 (1.3)	1 (2.6)
	Ear pruritus	1 (0.8)	0	1 (2.0)	0	1 (1.6)	1 (1.3)	0
	Hearing impaired	1 (0.8)	1 (1.4)	0	0	1 (1.6)	0	1 (2.6)
Gastrointestinal disorders		6 (5.0)	5 (7.1)	1 (2.0)	2 (3.4)	4 (6.6)	4 (5.0)	2 (5.1)
	Abdominal discomfort	2 (1.7)	2 (2.9)	0	0	2 (3.3)	2 (2.5)	0
	Diarrhoea	1 (0.8)	0	1 (2.0)	0	1 (1.6)	1 (1.3)	0
	Dry mouth	1 (0.8)	1 (1.4)	0	1 (1.7)	0	0	1 (2.6)
	Nausea	1 (0.8)	1 (1.4)	0	0	1 (1.6)	0	1 (2.6)
	Peptic ulcer	1 (0.8)	1 (1.4)	0	1 (1.7)	0	1 (1.3)	0
	Vomiting	1 (0.8)	1 (1.4)	0	0	1 (1.6)	0	1 (2.6)
General disorders and administration site conditions		27 (22.7)	15 (21.4)	12 (24.5)	13 (22.4)	14 (23.0)	20 (25.0)	7 (17.9)
	Asthenia	1 (0.8)	1 (1.4)	0	0	1 (1.6)	1 (1.3)	0
	Injection site pain	20 (16.8)	10 (14.3)	10 (20.4)	8 (13.8)	12 (19.7)	14 (17.5)	6 (15.4)
	Injection site swelling	3 (2.5)	1 (1.4)	2 (4.1)	0	3 (4.9)	3 (3.8)	0
	Pain	1 (0.8)	1 (1.4)	0	1 (1.7)	0	1 (1.3)	0
	Pyrexia^a^	6 (5.0)	4 (5.7)	2 (4.1)	5 (8.6)	1 (1.6)	5 (6.3)	1 (2.6)
Infections and infestations		3 (2.5)	1 (1.4)	2 (4.1)	2 (3.4)	1 (1.6)	3 (3.8)	0
	Abscess	1 (0.8)	0	1 (2.0)	1 (1.7)	0	1 (1.3)	0
	Ear infection	1 (0.8)	0	1 (2.0)	0	1 (1.6)	1 (1.3)	0
	Nasopharyngitis	1 (0.8)	1 (1.4)	0	1 (1.7)	0	1 (1.3)	0
Metabolism and nutrition disorders		1 (0.8)	0	1 (2.0)	0	1 (1.6)	1 (1.3)	0
	Malnutrition	1 (0.8)	0	1 (2.0)	0	1 (1.6)	1 (1.3)	0
Musculoskeletal and connective tissue disorders		1 (0.8)	0	1 (2.0)	1 (1.7)	0	0	1 (2.6)
	Neck pain	1 (0.8)	0	1 (2.0)	1 (1.7)	0	0	1 (2.6)
Nervous system disorders		3 (2.5)	1 (1.4)	2 (4.1)	0	3 (4.9)	2 (2.5)	1 (2.6)
	Burning sensation	1 (0.8)	0	1 (2.0)	0	1 (1.6)	1 (1.3)	0
	Tetanus	1 (0.8)	0	1 (2.0)	0	1 (1.6)	1 (1.3)	0
	Tinnitus	1 (0.8)	1 (1.4)	0	0	1 (1.6)	0	1 (2.6)
Psychiatric disorders		1 (0.8)	1 (1.4)	0	1 (1.7)	0	0	1 (2.6)
	Insomnia	1 (0.8)	1 (1.4)	0	1 (1.7)	0	0	1 (2.6)
Renal and urinary disorders		1 (0.8)	1 (1.4)	0	0	1 (1.6)	0	1 (2.6)
	Renal impairment	1 (0.8)	1 (1.4)	0	0	1 (1.6)	0	1 (2.6)
Respiratory, thoracic and mediastinal disorders		2 (1.7)	2 (2.9)	0	1 (1.7)	1 (1.6)	2 (2.5)	0
	Cough	2 (1.7)	2 (2.9)	0	1 (1.7)	1 (1.6)	2 (2.5)	0
Skin and subcutaneous tissue disorders		5 (4.2)	3 (4.3)	2 (4.1)	2 (3.4)	3 (4.9)	2 (2.5)	3 (7.7)
	Hyperhidrosis	3 (2.5)	2 (2.9)	1 (2.0)	2 (3.4)	1 (1.6)	0	3 (7.7)
	Pruritus	2 (1.7)	1 (1.4)	1 (2.0)	0	2 (3.3)	2 (2.5)	0

AE, adverse event.

Subjects with multiple occurrences of the same event are counted only once for a specific system organ class and preferred term.

^a^ One event of pyrexia reported in an adult male at site 2 was not treatment emergent.

Four subjects had 6 serious adverse events during the study (tinnitus, hearing impairment, and renal impairment all in one subject; and peptic ulcer, ear infection, and tetanus each in one subject). Peptic ulcer considered unrelated to study drug was reported in a pediatric male after EOT. Ear infection associated with slight to mild hearing loss considered possibly related to study drug was reported in an adult female after 4 doses of PMIM; the subject was withdrawn from study drug per protocol and improved after antibiotic treatment. Tinnitus, hearing impairment, and renal impairment, all considered possibly related to study drug, were reported in one adult male after EOT. The male subject also had a life-threatening adverse event of anemia, which is an expected manifestation of impaired renal function. The subject was treated, reported feeling better, and did not request further follow-up. Tetanus associated with severe malnutrition was reported after EOT in an adult female who subsequently died 22 days after EOT after being “lost against medical advice.” Both the tetanus and malnutrition were considered severe, but neither event was considered related to study drug.

No pregnancies were reported during the study treatment period. Pregnancy was reported in one female during the follow-up period. The offspring was born healthy, and a hearing test conducted on the infant at 1.5 months of age confirmed reaction to sound. An Otoscopy and Oto-Acoustic Emission test to determine function of the middle and inner ear was conducted at 3 months of age and confirmed normal hearing function.

## Discussion

In a Phase III randomized, controlled, open-label clinical trial in 667 children and adults in India, PMIM (11 mg/kg/day [base] administered intramuscularly for 21 days) was determined to be a safe and effective for treatment of VL [4]. PMIM was subsequently registered for the treatment of VL in India in 2006 and was included in the WHO Essential Medicines List in 2007.

In the absence of safe, effective and affordable treatment options, VL continues to remain an important parasitic disease in Bangladesh, and safe, effective, affordable treatments are needed. Results of this open-label, multi-center, single-arm, Phase IIIb clinical trial show the efficacy, safety, and compliance of the same regimen of PMIM (11 mg paromomycin base/kg/day for 21 days) for treatment of VL in rural areas of Bangladesh and support the use of PMIM as part of a combination therapy regimen. High and consistent response rates were observed across the initial (end of treatment) and final (6 months after end of treatment) assessments in this study. The final clinical response rate (94.2%) is similar to the final clinical response rate observed in the Phase III (94.2% final response) and Phase IV (94.6% final response) studies of PMIM in India [4,5].

The compliance rate of 98.3% in this study is similar to the 98% compliance rate in the Phase IV study conducted in 506 children and adults in an outpatient setting in India [5]. These high compliance rates in rural outpatient treatment settings further supports the use of PMIM in rural endemic areas of Bangladesh.

The evaluation based on clinical outcome at 6 months was planned for several reasons. This study was conducted with limited resources and was designed similar to a phase 4 study to generate evidence that PMIM works in Bangladesh similar to India, where the etiological agent and patient populations are thought to be very similar. The 6-month observation after treatment was the only practically feasible way to assess a cure in this study because splenic aspiration or other invasive procedures were not appropriate to conduct at the clinical study centers.

Adverse events were reported more frequently in females versus males and slightly more frequently in adults than in pediatrics (Tables 3 and 4). Few adverse events were reported by more than 1 subject. Injection site pain, which is an expected side effect of PMIM administration, was the most commonly reported adverse event. There was no involvement of the scitic nerve, which was found in cases of severe malaria treated with IM quinine in Africa [7]. Paromomycin belongs to the aminoglycoside class of agents, which are known to cause nephrotoxicity and ototoxicity. Patients with clinically significant renal dysfunction or history of hearing loss were excluded from this study. Nephrotoxicity and ototoxicity were not formally studied in this trial. During the study, 2 subjects reported adverse events related to hearing loss after end of treatment: moderate ear pruritus in an adult female and moderate hearing impairment and tinnitus in an adult male who also had moderate renal impairment. All of these events were reversible.

In conclusion, PMIM was found to be an effective and safe treatment for VL in Bangladesh. Compliance was high (98.3%) in this rural outpatient setting in Bangladesh. The short treatment duration and lower cost compared with other treatment options may make PMIM a preferred drug to include as part of a combination therapy regimen for VL patients in Bangladesh. Use of PMIM as part of combination therapy may reduce duration of treatment and reduce the chances of drug resistance [8] and is in line with recommendations by the Regional Technical Advisory Group of WHO for South East Region. This study supports the registration of PMIM for use in government health facilities in Bangladesh.
